# Supplementary material for: Do High Doses of Multiple Antibiotics Loaded into Bone Cement Spacers Improve the Success Rate in Staphylococcal Periprosthetic Joint Infection When Rifampicin Cannot Be Employed?
Source: Antibiotics (Basel). 2024 Jun 10;13(6):538. doi: 10.3390/antibiotics13060538 (PMC11200406; doi:10.3390/antibiotics13060538)
Supplement: Supplementary file 1 [file antibiotics-13-00538-s001.zip › antibiotics-2976825-Annex 1.pdf]

Annex 1. Characteristics, systemic antibiotic treatment, and outcomes of patients who did not receive an oral rifampicin-containing regimen.

N.	Age, years	Gender	Index Surgery		Microorganism	TKY	Antimicrobial regimen	Length, weeks	Spacer exchange	Cultures/ Sonication	Microorganism	Follow-up time/TTF	Outcome
1	75	F	THA	R	MSSA / P (*P mirabilis*)	III	Cotrimoxazole + Fosfomycin	24	No	1 +	Trichosporon asahii (contaminant)	182	Cure
2	59	F	HHA	P	MRSE/ P (*C albicans*)	II	Daptomycin + Fluconazole	11	No	Negative		200	Reintervention (resection
3	75	F	THA	P	MSSE	IV	Cotrimoxazole	6	Dislocation	Negative		113	Cure
4	66	F	THA	P	MSSA	IV	Cotrimoxazole + Levofloxacin	20	No	3 +	MRSE	150	Cure
5	84	F	TKA	R	MSSA /MRSE	IV	Linezolid + Fosfomycin	6	No	Negative		32	Cure
6	72	F	TKA	P	MSSA	II	Cotrimoxazole + Levofloxacin	21	No	Negative		82	Cure
7	79	F	TKA	P	MSSA	III	Cefazolin + Levofloxacin + Daptomycin	5	No	Negative		280	Cure
8	67	F	TKA	P	MRSE / SL / P (*P mirabilis*)	IV	Cotrimoxazole + Ciprofloxacin	14	No	Negative		38	Cure
9	81	F	THA	R	MSSA	IV	Cotrimoxazole + Levofloxacin + Cefazolin	23	No	1 +	MSSE (contaminant)	42	Cure
10	75	F	THA	P	MRSE	II	Daptomycin + Levofloxacin	6	No	Negative		22	Cure
11	75	F	TKA	P	MSSE / P (*Salmonella, Micrococcus luteus*)	IV	Cotrimoxazole + Ciprofloxacin + Amoxicillin-clavulanic acid	6	No	Negative		20	Cure
12	69	F	THA	R	MRSE	IV	Cotrimoxazole + Levofloxacin	12	No	2 +	MSSA	18	Cure
13	57	M	THA	R	SL	IV	Levofloxacin + Amoxicillin-clavulanic acid	12	No	Negative		14	Cure
14	70	M	TKA	P	MSSA	II	Daptomycin + Meropenem	8	No	Negative		20	Cure
15	71	M	TKA	P	MSSA	III	Cotrimoxazole + Ciprofloxacin	8	No	Negative		24	Cure
16	72	M	THA	R	MRSE	II	Cotrimoxazole + Levofloxacin	6	No	Negative		30	Cure
17	73	M	TKA	P	MRSE / P (*E feacalis*)	I	Tedizolid	8	No	Negative		64	Reintervention (revision
18	74	M	THA	P	MSSA / P (*E cloacae*)	II	Levofloxacin + Fosfomycin	9	No	Negative		18	Cure
19	75	M	THA	P	MSSE / P (*H parainfluenzae*)	II	Levofloxacin + Amoxicillin-clavulanic acid	8	Infection persistance	Negative		150	Reintervention (revisión)
20	76	M	TKA	T	MRSE / P (*C parapsilosis*)	IV	Daptomycin + Fluconazole + Doxycyclin	S	No	3 +	MRSE, candida parapsilosis,	57	Supressive treatment
21	77	M	TKA	T	MRSE	IV	Daptomycin + Linezolid	12	No	Negative		61	Cure
22	78	M	TKA	T	MSSE	II	Linezolid + Daptomycin / Ciprofloxacin + Meropenem	21	No	2 +	MRSE, multiresistant pseudomonas aureginosa	133	Amputation
23	79	M	TKA	P	MRSE	IV	Cotrimoxazole + Levofloxacin	8	Infection persistance	Negative		21	Cure
24	80	M	TKA	R	MSSE	IV	Daptomycin + Linezolid	8	Infection persistance	Negative		22	Cure

TKY = Tsukuyama; TTF = time to failure; THA = total hip arthroplasty; TKA = total knee arthroplasty; HHA= hip hemiarthroplasty; P = primary; R = revision; T = tumoral reconstructive surgery; MRSA: Methicillin resistant Staphylococcus aureus, MSSA = Methicillin sensitive Staphylococcus aureus; MRSE = Methicillin resistant Staphylococcus epidermidis; MSSE = Methicillin sensitive. Staphylococcus epidermidis; CoNS = Coagulase-negative staphylococci; SL = Stapylococcus lugdunensis; P =Polymicrobial infection; TTF = time to failure.
