# Supplementary material for: Do High Doses of Multiple Antibiotics Loaded into Bone Cement Spacers Improve the Success Rate in Staphylococcal Periprosthetic Joint Infection When Rifampicin Cannot Be Employed?
Source: Antibiotics (Basel). 2024 Jun 10;13(6):538. doi: 10.3390/antibiotics13060538 (PMC11200406; doi:10.3390/antibiotics13060538)
Supplement: Supplementary file 1 [file antibiotics-13-00538-s001.zip › antibiotics-2976825-Annex 2.pdf]

Annex 2

Biopsy specimens were aseptically disrupted in sterile mortar with saline solution. Aliquots of 100 µl of synovial fluid were cultured on sheep blood, chocolate, and brucella agar for 2 days at 37°C in air, in 5% CO_2, and anaerobically, respectively. Chocolate and brucella agar plates were reincubated for up to 7 days. Samples were also cultured in brain heart infusion broth for 10 days and subcultured when turbid. Gram stain was performed on all samples.
The bacteria isolated were identified and susceptibility testing was performed using MicroScan panels (Siemens Healthcare Diagnostic, West Sacramento, CA) and conventional microbiological procedures according to CLSI recommendations.
